# Guillain–Barre Syndrome—A Rare Cause of Quadriparesis after the Bentall Procedure for Type A Aortic Dissection

**DOI:** 10.1055/s-0042-1756668

**Published:** 2022-12-15

**Authors:** Rajeev T. Chellasamy, Aravind Kalyanasundaram, Hemachandren Munuswamy, Ramkumar Sugumaran, Rajesh K. Meher

**Affiliations:** 1 Departments of Cardiothoracic and Vascular Surgery, Jawaharlal Institute of Postgraduate Medical Education and Research, Puducherry, India; 2 Department of Neurology, Jawaharlal Institute of Postgraduate Medical Education and Research, Puducherry, India

**Keywords:** Guillain–Barre syndrome, cardiac surgery, aortic dissection, quadriparesis

## Abstract

Neurological complications following aortic surgery are most often cerebrovascular accidents due to embolism, or spinal infarcts resulting in hemiparesis or hemiplegia. Guillain–Barre syndrome is a rare cause of quadriparesis. Here, we report a 49–year old male who presented with acute aortic dissection and underwent the Bentall procedure following which he developed quadriparesis, subsequently diagnosed to be a case of Guillain–Barre syndrome. He was successfully treated with intravenous immunoglobulin.

## Introduction

Neurological complication following aortic surgery ranges from focal neurological deficit to coma. Postoperative quadriparesis following the Bentall surgery for Type A aortic dissection is very rare. Neurological complications can occur due to cardiopulmonary bypass (CPB), which can cause to emboli, cerebral hypoperfusion, or hypotension. 1 Other causes of neurological injury include spinal ischemia following thrombosis of the false lumen after the surgery. Guillaume–Barre syndrome (GBS) following major surgery has been reported, but there are very few reports in the literature of GBS following the Bentall procedure. 2

## Case Presentation

**Fig. 1:**
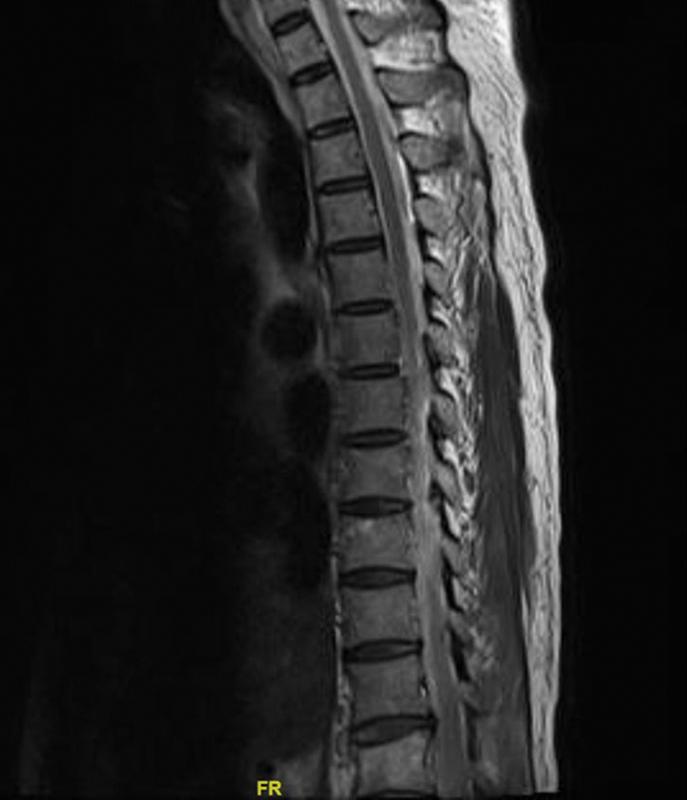
Postoperative magnetic resonance imaging spine which was normal.

A 49-year-old male patient presented with sudden onset of chest pain radiating to the back, not associated with sweating. He was conscious, oriented, and did not have any sensory or motor deficit. ECG showed a left ventricular (LV) hypertrophy pattern, and there were no features of acute myocardial infarction. Troponin was negative. Echocardiogram showed severe aortic regurgitation with concentric LV hypertrophy. The aortic valve was tricuspid, and LV function was normal. A dissection flap was seen in the ascending aorta. Computed tomographic aortography confirmed Type A dissection, extending from the ascending aorta to the aortic bifurcation. The dissection flap was seen extending into the base of the innominate artery and up to the right carotid artery. The innominate artery, left carotid, and left subclavian arteries were seen arising from true lumen. All the visceral arteries were arising from the true lumen. The patient was taken to the operating room for the Bentall procedure. The left carotid artery and right axillary artery were exposed. Near-infrared spectroscopy (NIRS) monitoring was utilized. The baseline value was 65% on both sides. Median sternotomy was done. CPB was established via the right axillary artery, with superior and inferior vena cava cannulation. An LV vent was placed through the right superior pulmonary vein. Core cooling was done. The aortic cross-clamp (ACC) was applied, and the dissected ascending aorta was opened. The heart was arrested via direct coronary ostial del Nido cardioplegia. A composite valved graft was prepared with a 23-mm TTK Chitra aortic valve and a 26-mm Dacron graft. The dissected ascending aorta and aortic valve were replaced with this composite aortic valved conduit. Core temperature was reduced to 24°C. The innominate artery, left carotid, and left subclavian artery were snugged, circulatory arrest was instituted, and antegrade cerebral perfusion (ACP) was maintained through the right carotid artery. Since the NIRS dropped below 55%, the left carotid artery was exposed and an ACP cannula was inserted. ACP was now maintained through both carotid arteries. NIRS improved gradually. Total circulatory arrest was initiated. ACC was released. The proximal arch was prepared with Teflon felt. A 26-mm vascular graft was anastomosed to the proximal arch. The neograft was clamped, and distal perfusion was established through the innominate artery. Blood cardioplegia was given intermittently every half an hour after 90 minutes of arrest following del Nido cardioplegia. The circulatory arrest time was 30 minutes. Rewarming was initiated, and the heart was deaired. The patient was gradually weaned off CPB. Total CPB time was 6 hours 15 minutes, and ACC time was 3 hours 25 minutes. The patient was moved to intensive care unit in a hemodynamically stable state. On postoperative days 1 and 2, the patient was disoriented and confused but was moving all four limbs. On postoperative day 4, he started regaining consciousness and was extubated. On postoperative day 7, we noticed that he had weakness of both lower limbs with preserved upper limb movement. Then, within a period of 1 day, he developed weakness of the upper limbs as well. He had no fever during the entire course. A neurological opinion was sought. On examination, he had flaccid quadriparesis and absent reflexes, with intact sensation. Bladder function could not be assessed as he was catheterized, but he was able to feel a bladder sensation when his urinary catheter was clamped. Magnetic resonance imaging of the whole spine, with brain screening as well, was normal, revealing no evidence of spinal infarct ( Fig. 1 ). Nerve conduction study showed reduced sensory and motor conduction velocity with prolonged distal latency period and F-wave latency, with preserved sensory and motor amplitudes in both upper and lower limbs, suggestive of demyelination. Based upon the above clinical, neuroimaging, and electrophysiological picture, he was diagnosed as a case of GBS. We initially started him on plasmapheresis, but he had repeated episodes of hypotension. Plasmapheresis was stopped on the same day. Intravenous immunoglobulin was started. A total dosage of 100 g (20 g/d) was given over a period of 5 days. The patient improved clinically over a period of 10 days and was able to walk independently. He was followed-up after 8 months when he had no complaints.

## Discussion

GBS is a postinfectious disorder, and two-thirds of adult patients report preceding symptoms of a respiratory or gastrointestinal tract infection within 4 weeks of the onset of weakness. 3 It is challenging to diagnose a surgery-related GBS, as such cases are often underestimated. According to a French registry-based large nationwide epidemiological study, GBS was found to be moderately associated with any recent surgery and was more strongly related to orthopaedic and abdominal surgeries. 4 GBS following cardiac surgery was found to account for only 16.1% of instances in a case series by Nagarajan et al. 5 Their study demonstrated a close relationship between comorbid autoimmune disease or malignancy and the development of postsurgical GBS. However, there was no report of GBS following a Bentall procedure in that study. Raut et al 6 reported a case of GBS following surgery for the ruptured sinus of Valsalva. The possible mechanism may be due to postsurgical stress causing systemic immune response against myelin. Surgery has been thought to alter the balance of the immune system, leading to transient immunosuppression, possibly acting as a trigger for GBS development. 7 There is no consensus about the association between postsurgical GBS and malignancy or preexisting autoimmune disease. This needs to be a direction for future research. In postsurgical GBS, the axonal subtype is more common than the demyelinating subtype. 8 But in our case, the nerve conduction study showed a demyelinating pattern. Treatment for GBS includes plasma exchange and immunoglobulin therapy. Our patient showed prompt improvement in muscle strength after initiation of intravenous immunoglobulin.

We present this case because GBS following cardiac surgery is rare, and it can confuse the treating surgeon, masquerading as typical neurological complications following cardiac surgery, secondary to embolism or hypotension. But, in most scenarios standard cerebrovascular complications occur in the immediate postoperative period, soon after the completion of the cardiac surgery. In contradistinction, our patient weakness appeared late, after a week. Such timing may guide the treating surgeon to look for other causes. In the setting of late-onset weakness, GBS should be considered as a possibility following cardiac surgery.

GBS is a rare cause of postoperative quadriparesis after major surgery and should be considered in the differential diagnosis, especially if there is delayed onset of symptoms. Intravenous immunoglobulin and plasma exchange are equally effective in the management of GBS, and they should be started immediately after the diagnosis. Delayed treatment will not be effective.
